# On the recurrent neural network model with robust expectile-based loss function in economic data forecasting

**DOI:** 10.1016/j.mex.2025.103718

**Published:** 2025-11-10

**Authors:** Wisnowan Hendy Saputra, Rinda Nariswari, Matthew Owen

**Affiliations:** a Computer Science Department, School of Computer Science, Bina Nusantara University, Jakarta, 11530, Indonesia; b Statistics Department, School of Computer Science, Bina Nusantara University, Jakarta, 11530, Indonesia

**Keywords:** Expectile, Recurrent neural network, Long-short term memory, Gated recurrent unit, Economic forecasting

## Abstract

Recurrent Neural Networks (RNNs), particularly their Long Short-Term Memory (LSTM) and Gated Recurrent Unit (GRU) variants, are standard methods for modeling sequential data. However, their robustness is often limited when faced with non-stationary and heterogeneous time series data. This limitation is largely due to their reliance on symmetric loss functions such as mean squared error, which implicitly assume homogeneous data patterns. To address this, we propose a new framework, Expectile-based Recurrent Neural Network (E-RNN), which integrates expectile regression into the RNN architecture. We implement and compare two E-RNN variants, namely E-LSTM and E-GRU, to obtain the best forecast. . By leveraging the asymmetric least squares loss function, the E-RNN model is able to model various parts of the conditional data distribution, not just its central tendency. This allows forecasting across scenarios, ranging from pessimistic to optimistic, by adjusting the asymmetric parameter (*τ*), a value within the range (0, 1) where *τ*〈 0.5 yields pessimistic and *τ*〉 0.5 yields optimistic forecasts.. We demonstrate this methodology by forecasting Indonesia's quarterly economic growth data from 2001 to 2025. Empirical results show that the E-RNN model consistently exhibits superior performance, evidenced by lower Expectile-based Generalized Approximate Cross Validation (EGACV) scores for model selection and higher forecast accuracy. This superiority becomes particularly significant on more volatile quarter-to-quarter (qtq) data, highlighting the effectiveness of this framework in adapting to complex data dynamics and improving forecast reliability under uncertain conditions.

• Integrates expectile properties into RNN architectures to create models that are adaptive to changes in data distribution and are not tied to the homogeneity assumption.

• Introduces a robust model selection criterion: Expectile-based Generalized Approximate Cross Validation (EGACV). This criterion effectively balances model fit with complexity within an expectile framework..

• Generates a set of forecasts for various outcome scenarios (e.g., pessimistic, optimistic) by adjusting a single asymmetric parameter (τ), moving beyond single-point estimation.

## Graphical abstract

**Figure:**
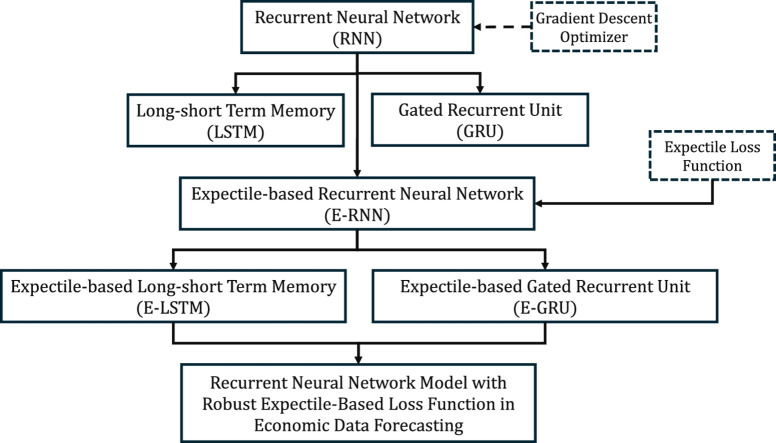

**Specifications table**

**Table:** 

**Subject area**	Computer Science
**More specific subject area**	Machine Learning for Forecasting
**Name of your method**	Expectile-based Recurrent Neural Network
**Name and reference of original method**	Developed based on expectile regression applied to Recurrent Neural Network, including Long-short Term Memory and Gated Recurrent Unit
**Resource availability**	N/A

## Background

Recurrent Neural Network (RNN) is a type of artificial neural network designed to handle sequential data, such as time series data. Its ability to store and process information from previous steps makes RNN a very useful tool in analyzing and forecasting time-based data. The versatility of Artificial Neural Networks (ANNs) and their variants, such as RNNs, is not limited to economics; they have become indispensable tools in diverse scientific and engineering fields. For instance, ANNs have been successfully applied to optimize complex systems in fluid dynamics [1], analyze the reliability of engineering components in comparison to traditional statistical methods like Maximum Likelihood Estimation [2], and even model survival data in disease analysis [3]. Their ability to capture complex non-linear patterns makes them exceptionally well-suited for problems where traditional models may struggle. In time series forecasting, RNNs learn temporal patterns from historical data to predict future values, such as stock price movements, energy consumption, or product demand. RNNs process data incrementally, one point in time at a time, while updating its hidden state to capture temporal relationships in the data [4]. However, while effective in capturing short-term patterns, RNNs have limitations in handling long-term dependencies due to issues such as vanishing gradient [[5], [6], [7]]. To overcome this, RNN variants such as Long Short-Term Memory (LSTM) and Gated Recurrent Unit (GRU) were developed [8]. Both architectures are designed to retain important information from the past over a longer period of time, thus enabling more accurate predictions, even for data that is complex and has significant seasonal patterns or trends [9]. With the ability to understand temporal patterns and make predictions based on past data, RNNs remain one of the important foundations in forecasting time series data and continue to be the subject of development in the field of machine learning.

LSTM is one of the variants of RNN designed to overcome the limitations of standard RNN in handling long-term dependencies on sequential data. The LSTM architecture consists of three main components called input gate, forget gate, and output gate. The input gate determines which new information will be added to the cell state; the forget gate governs which information from the past should be forgotten; and the output gate controls which information will be used to generate the output at the current time step [10]. The combination of these mechanisms allows the LSTM to capture long-term patterns and avoid losing important information from historical data [11]. GRU is another variant of RNN developed to address long-term dependency issues such as vanishing gradient. Like LSTM, GRU is designed to capture long-term patterns in sequential data. However, GRU has a simpler architecture compared to LSTM, with the aim of improving computational efficiency without sacrificing the ability to process temporal relationships in the data [12]. GRU combines the forget gate and input gate functions in LSTM into a single component called the update gate [13]. In the context of forecasting time series data, GRU has the ability to learn seasonal patterns and trends from historical data in a similar way to LSTM [14]. This model is widely used in applications such as market demand prediction, stock price movements, or weather data analysis [[15], [16], [17]]. Its lighter nature makes GRU an ideal choice for applications where speed is a priority, without significantly sacrificing prediction accuracy [18].

While RNNs have proven to be a powerful method, their fundamental limitation lies in the symmetric loss functions (e.g., Mean Squared Error) they employ. Such loss functions implicitly train the model to predict the conditional mean, which assumes that data patterns are homogeneous [19,20]. This assumption often fails for heterogeneous and non-stationary economic data, where outliers and skewed distributions make the mean an unstable and unrepresentative statistic. As a result, conventional RNNs lack robustness when faced with dynamic data shifts or structural breaks [21]. To overcome this limitation, expectile regression-based approaches offer a more robust solution [[22], [23], [24]]. Instead of targeting the fragile mean, expectile regression uses an asymmetric loss function capable of modeling different parts of the data’s conditional distribution. By assigning different penalty weights to overestimation and underestimation errors, the model can be guided to capture smoother dynamics or asymmetric risk scenarios [25,26]. This flexibility is particularly useful for data with high variability or outliers, allowing the model to capture the dynamics of a wider distribution and adapt to changing data patterns [27].

By integrating the concept of expectile regression into the RNN architecture, the model is not only able to handle temporal dependence but also more adaptive to changes in data distribution. Thus, we propose a new Expectile-based Recurrent Neural Network (E-RNN) model. This model makes the RNN more robust, as it is no longer bound to the assumptions of homogeneity or normal distribution. Thus, the expectile-based solution opens up opportunities to extend the capabilities of RNNs in handling complex and non-stationary data. This innovation also reflects the growing effort to improve the robustness of machine learning models, making them more relevant for real-world applications where data often does not follow a stable or uniform pattern. The expectile regression approach integrated in the RNN architecture makes the estimation process used must utilize an Asymmetric Least Squares (ALS) based loss function. This loss function modifies the quantile loss function by changing the L1-norm to L2-norm so that it is differentiable and can use estimation methods through standard gradient-based numerical iteration. The use of RNN architecture in the model should also consider the best model selection scheme that follows the model hyperparameter combination scheme. Model hyperparameters are defined to consist of the number of nodes in the layer, the number of hidden layers, and the expectile value. To obtain the best model combination, inspired by Yuan [28], the selection is based on the expectile-based Generalized Aproximate Cross Validation (GACV) value which is then referred to as Expectile-based GACV (EGACV). Finally, validation of the forecast results is done using the Asymmetric Mean Absolute Percentage Error (AMAPE) and Asymmetric Root Mean Square Error (ARMSE) measures.

## Method details

**Fig. 1:**
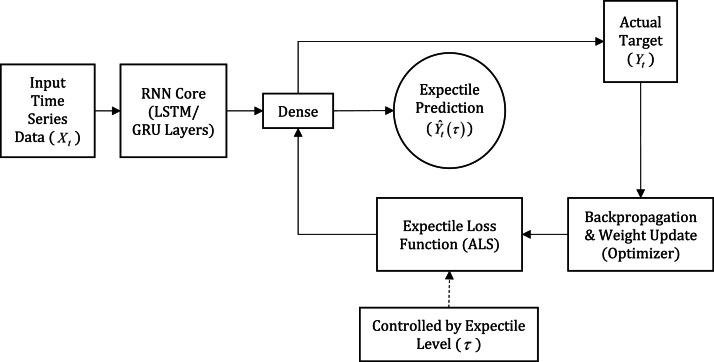
Conceptual Schematic Diagram of the Expectile-based Recurrent Neural Network (E-RNN) Architecture.

Recurrent Neural Network (RNN) is a type of artificial neural network developed to process sequential data. The basic concept of RNN emerged from the initial development of artificial neural networks in 1982 by John Hopfield in his research entitled Neural Network and Physical Systems with Emergent Collective Computational Abilities. Hopfield [29] developed the RNN model through the idea that computers could emerge from systems consisting of many simple components such as neurons. The concept of memory that can be accessed based on content is explained by looking at how the state of the system changes in phase space. The RNN model is based on neurobiology but can also be applied to electronic circuits. RNN models are capable of generating a complete memory from just a small portion of data, as long as it is large enough. It uses parallel processing in an asynchronous manner. In addition, it has other capabilities such as generalization, recognizing familiar patterns, clustering, error correction, and storing time sequences. The proposed methodology, Expectile-based Recurrent Neural Network (E-RNN), is designed to improve the robustness of RNN models in handling non-stationary and heterogeneous time series data. This is achieved by integrating expectile regression properties into the RNN architecture. This section details the model architecture, estimation process, model selection scheme, and evaluation metrics used, with an emphasis on its implementation on popular RNN variants: Long Short-Term Memory (LSTM) and Gated Recurrent Unit (GRU). These two variants are developed into E-LSTM and E-GRU for performance comparison. The E-RNN architecture utilizes sequential processing capabilities of RNN models such as LSTM and GRU but modifies its learning objective. To clarify the integration of expectile regression into the E-RNN architecture, Fig. 1 presents a conceptual schematic diagram. This diagram shows how the expectile approach does not change the internal structure of the recurrent unit (such as an LSTM or GRU), but instead modifies the output layer and loss function to produce expectile predictions (${\hat{Y}}_{t}\left(\tau \right)$) instead of the conventional average prediction.

Instead of predicting the mean value, the E-RNN is trained to predict the τ-th conditional expectile of the target variable. E-RNN models can be built using one of two main recurrent architectures as a backbone to capture temporal dependencies, the first using a basic long-short term memory (LSTM) model and the second using a gated recurrent unit (GRU).

### Expectile-based long-short term memory

This model uses Long Short-Term Memory (LSTM) cells as hidden processing units. LSTM cells are specifically designed to address the vanishing gradient problem common in traditional RNNs, allowing the model to retain information over longer periods of time [30,31]. Each LSTM cell has a complex internal architecture consisting of three gates [29]:

■ Forget Gate ($F_{g}$): Controls what information from the previous cell state should be forgotten.
■ Input Gate ($I_{g}$): Controls what new information will be added to the cell state.
■ Output Gate ($O_{g}$): Determines the hidden state output value based on the current cell state.

These gates use a sigmoid activation function to generate a value between 0 and 1, which controls the flow of information. The final hidden state ($H_{t}$) from the LSTM layer (or stack of LSTM layers) is then passed to the output layer (usually a dense layer), which is no longer activated to generate an average prediction, but rather to generate an expectile prediction ${\hat{Y}}_{t}^{\left(\tau \right)}$, so the called Expectile-based LSTM (E-LSTM). The entire network, including the weight parameters of the E-LSTM gates ($W_{f}^{\left(\tau \right)},W_{i}^{\left(\tau \right)},W_{o}^{\left(\tau \right)}$) and the output layer, is optimized using the expectile loss function.

Mathematically, suppose there are h hidden units, the batch size is n, and the number of inputs is d. Then, the input is defined as $X_{t}\in R^{n\times d}$ and the hidden state of the previous time step is $H_{t-1}^{\left(\tau \right)}\in R^{n\times d}$. Thus, the gates at time step t are defined as follows: the input gate is $I_{t}^{\left(\tau \right)}\in R^{n\times d}$, the forget gate is $F_{t}^{\left(\tau \right)}\in R^{n\times d}$, and the output gate is ${\hat{Y}}_{t}^{\left(\tau \right)}\in R^{n\times d}$. The gates mentioned are calculated using the following equation:

(2.1) $$I_{t}^{\left(\tau \right)}=\sigma \left(X_{t}W_{xi}^{\left(\tau \right)}+H_{t-1}^{\left(\tau \right)}W_{hi}^{\left(\tau \right)}+b_{i}^{\left(\tau \right)}\right),$$

(2.2) $$F_{t}^{\left(\tau \right)}=\sigma \left(X_{t}W_{xf}^{\left(\tau \right)}+H_{t-1}W_{hf}^{\left(\tau \right)}+b_{f}^{\left(\tau \right)}\right),$$

(2.3) $${\hat{Y}}_{t}^{\left(\tau \right)}=O_{t}^{\left(\tau \right)}=\sigma \left(X_{t}W_{xo}^{\left(\tau \right)}+H_{t-1}^{\left(\tau \right)}W_{ho}^{\left(\tau \right)}+b_{o}^{\left(\tau \right)}\right),$$

where $W_{xi}^{\left(\tau \right)},\;W_{xf}^{\left(\tau \right)},\;W_{xo}^{\left(\tau \right)}\in R^{d\times \;h}$ and $W_{hi}^{\left(\tau \right)},\;W_{hf}^{\left(\tau \right)},\;W_{ho}^{\left(\tau \right)}\in R^{h\;\times \;h}$ are weight parameters and $b_{i}^{\left(\tau \right)},\;b_{f}^{\left(\tau \right)},\;b_{o}^{\left(\tau \right)}\in R^{1\;\times \;h}$ are bias parameters, which all depends on the value of a particular τ-expectile with 0<τ<1. Next, the E-LSTM model parameters are estimated by minimizing an empirical Asymmetric Least Squares (ALS) loss function, also referred to as the expectile loss function, to obtain robust estimates. Mathematically, the minimization for E-LSTM model parameter estimation is written as follows:
  (2.4) $$L_{E-LSTM}^{\left(\tau \right)}\left(W_{xo}^{\left(\tau \right)},W_{ho}^{\left(\tau \right)},b_{o}^{\left(\tau \right)}\right)=\frac{1}{T}\sum_{t=1}^{T}\rho_{\tau }\left(Y_{t}-{\hat{Y}}_{t}^{\left(\tau \right)}\right),$$
where T denotes the number of observations, Y is the vector of target, and $\rho_{\tau }\left(\varepsilon \right)=\{\begin{array}{c} \left(1-\tau \right)\varepsilon^{2},\;\varepsilon <0\\ \tau \varepsilon^{2},\;\varepsilon \geq 0 \end{array}$.

### Expectile-based gated recurrent unit

The second RNN model developed is the Expectile-based Gated Recurrent Unit (E-GRU). This model uses the architecture of the standard Gated Recurrent Unit (GRU) model. GRU is a simpler RNN variant than LSTM but still effective in handling long-term dependencies [32,33]. GRU simplifies the LSTM architecture by using only two gates [29]:

■ Update Gate ($Z_{g}$): Combines the forget function and input gate of the LSTM, determining how much past information should be retained and how much new information should be added.
■ Reset Gate ($R_{g}$): Controls how much information from the previous hidden state is forgotten and how relevant that information is to the calculation of the current candidate hidden state.

Due to its lighter structure, GRU is generally computationally faster and requires fewer parameters than LSTM, making it an ideal choice for applications that prioritize speed without significantly sacrificing prediction accuracy. The final hidden state ($H_{t}$) from the GRU layer is passed to the output layer, where it is optimized to produce an expectile prediction$\;{\hat{Y}}_{t}^{\left(\tau \right)}$, so the called Expectile-based GRU (E-GRU).

Mathematically, suppose there are h hidden units, the batch size is n, and the number of inputs is d. Then, the input is defined as $X_{t}\in R^{n\times d}$ and the hidden state of the previous time step is $H_{t-1}\in R^{n\times d}$. Thus, the gates at time step t are defined as follows: the reset gate is $R_{t}\in R^{n\times d}$ and the update gate is $Z_{t}\in R^{n\times d}$. The gates mentioned are calculated using the following equation:

(2.5) $$R_{t}^{\left(\tau \right)}=\sigma \left(X_{t}W_{xr}^{\left(\tau \right)}+H_{t-1}^{\left(\tau \right)}W_{hr}^{\left(\tau \right)}+b_{r}^{\left(\tau \right)}\right),$$

(2.6) $$Z_{t}^{\left(\tau \right)}=\sigma \left(X_{t}W_{xz}^{\left(\tau \right)}+H_{t-1}^{\left(\tau \right)}W_{hz}^{\left(\tau \right)}+b_{z}^{\left(\tau \right)}\right),$$

where $W_{xr}^{\left(\tau \right)},\;W_{xz}^{\left(\tau \right)}\in R^{d\times \;h}$ and $W_{hr}^{\left(\tau \right)},\;W_{hz}^{\left(\tau \right)}\in R^{h\;\times \;h}$ are weight parameters and $b_{r}^{\left(\tau \right)},\;b_{z}^{\left(\tau \right)}\in R^{1\;\times \;h}$ are bias parameters, which all depends on the value of a particular τ-expectile with 0<τ<1. Next, the E-GRU model parameters are estimated by minimizing the Asymmetric Least Squares (ALS) loss function, defined in the following equation:

(2.7) $$L_{E-GRU}^{\left(\tau \right)}\left(W_{xr}^{\left(\tau \right)},\;W_{xz}^{\left(\tau \right)},b_{r}^{\left(\tau \right)},b_{z}^{\left(\tau \right)}\right)=\frac{1}{T}\sum_{t=1}^{T}\rho_{\tau }\left(Y_{t}-{\hat{Y}}_{t}^{\left(\tau \right)}\right),$$

where T, Y, and $\rho_{\tau }\left(\varepsilon \right)$ has the same explanation as Eq. (2.4).

The core innovation of the E-RNN model lies in the loss function used during training, not in the internal changes of the recurrent cells themselves. Thus, the basic architecture still leverages the power of LSTM/GRU in modeling temporal dependencies, while its output layer is tailored for expectile modeling purposes. Parameter estimation of E-RNN models (both E-LSTM and E-GRU) is performed by minimizing the ALS loss function. This function imposes different penalties for overestimation and underestimation errors, controlled by the expectile parameter (τ). This parameter is a tunable value within the range (0,1) that dictates the nature of the forecast. Specifically:

- τ<0.5 imposes a greater penalty on underestimation, leading to pessimistic forecasts.
- τ=0.5 applies a symmetric penalty, which is equivalent to predicting the mean.
- τ>0.5 imposes a greater penalty on overestimation, leading to optimistic forecasts.

In more detail, for one $\left(Y_{t},{\hat{Y}}_{t}^{\left(\tau \right)}\right)$ data point, the ALS loss function is defined as:

(2.8) $$\rho_{\tau }\left(Y_{t}-{\hat{Y}}_{t}^{\left(\tau \right)}\right)=v_{\tau }{\left(Y_{t}-{\hat{Y}}_{t}^{\left(\tau \right)}\right)}^{2},$$

where $v_{\tau }$ is an asymmetric weight function:

(2.9) $$v_{\tau }=\left\{ \begin{array}{c} \left(1-\tau \right),\;Y_{t}<{\hat{Y}}_{t}^{\left(\tau \right)}\\ \tau ,\;Y_{t}\geq {\hat{Y}}_{t}^{\left(\tau \right)} \end{array}\right..$$

The total loss function for the entire training set with T observations is:

(2.10) $$L_{ALS}^{\left(\tau \right)}=\frac{1}{T}\sum_{t=1}^{T}v_{\tau }{\left(Y_{t}-{\hat{Y}}_{t}^{\left(\tau \right)}\right)}^{2},$$

where τ represents all learnable parameters in the network (e.g., the weight matrices in the LSTM/GRU gates, biases, and weights in the output layer). ${\hat{Y}}_{t}^{\left(\tau \right)}$ is the expectile output of the E-RNN model at time t. This expectile loss function is differentiable, which is a major advantage over quantile (L1-norm-based) loss functions. This differentiability allows us to use efficient standard gradient-based optimization algorithms, such as Backpropagation Through Time (BPTT), to train the network. Modern optimization algorithms such as Adam, RMSprop, or Adagrad can be applied to iteratively update the τ parameters to minimize the $L_{ALS}^{\left(\tau \right)}$.

To strengthen the link between theory and application, it is essential to explain how the ALS loss function fundamentally alters the learning behavior of the RNN during training, especially when compared to a symmetric loss function like Mean Squared Error (MSE). In a conventional RNN using MSE, the loss for an observation is simply ${\left(Y_{t}-{\hat{Y}}_{t}\right)}^{2}$. During backpropagation, the gradient calculated from this loss is symmetric. This means a prediction error of +0.5 % (an underestimation) generates a gradient of the same magnitude (though opposite in direction) as an error of −0.5 % (an overestimation). Consequently, the weight updates consistently push the model's parameters to find a central balance point, which converges to the conditional mean. In contrast, the ALS loss function, $v_{\tau }{\left(Y_{t}-{\hat{Y}}_{t}^{\left(\tau \right)}\right)}^{2}$, introduces asymmetry into the learning process via the $v_{\tau }$ weight. Let us consider an intuitive example in the context of GDP forecasting:

- Scenario: Suppose we aim to generate a pessimistic forecast (e.g., for risk analysis) by setting τ=0.1. This implies the weight $v_{\tau }$ will be 0.9 for underestimation (when $Y_{t}>{\hat{Y}}_{t}^{\left(\tau \right)}$) and 0.1 for overestimation (when $Y_{t}\leq {\hat{Y}}_{t}^{\left(\tau \right)}$).
- Learning Behavior: If the actual GDP is 4 % and the model predicts 3 % (an underestimation), the calculated loss is heavily penalized by the 0.9 wt. The resulting gradient will be strong, pushing the model's weights to produce a higher prediction in the next iteration. However, if the model predicts 5 % (an overestimation), the loss is multiplied by a small 0.1 wt. The resulting gradient will be weak, signaling to the model that this type of error is less critical.

Over thousands of training iterations, this asymmetric behavior effectively "teaches" the model that underestimation is far more 'costly' than overestimation. As a result, the RNN's parameters converge not to the mean, but to a point where approximately 10 % of the observations lie below the forecast value—the definition of the 0.1-expectile. It is through this mechanism that the expectile loss function enables the RNN to systematically map the entire conditional distribution of the target variable, moving beyond a single-point mean forecast to provide richer, more informative predictions.

Selecting the best E-RNN model requires determining the optimal combination of hyperparameters. This process is crucial to prevent overfitting and ensure good generalization. The main hyperparameters to consider are: First, Network Architecture: Number of hidden layers and number of units (neurons) in each layer; Second, Expectile Level (τ): The most relevant τ value for the problem at hand; Third, Training Parameters: Learning rate, batch size, number of epochs, dropout rate, etc. To perform systematic model selection, we propose the use of Expectile-based Generalized Approximate Cross Validation (EGACV), adapted from Yuan [28]. The EGACV criterion provides a balance between goodness-of-fit to the training data and model complexity.

(2.11) $$EGACV=\frac{\sum_{t=1}^{T}v_{\tau }{\left(Y_{t}-{\hat{Y}}_{t}^{\left(\tau \right)}\right)}^{2}}{T-\text{df}},$$

where df is the effective degrees of freedom of the model, which serves as a penalty for model complexity. The model with the lowest EGACV value is considered the best model, as it exhibits the optimal balance between accuracy and complexity. The model selection procedure follows these steps: Define a grid search space for relevant hyperparameters; Train an E-RNN model for each combination of hyperparameters in the grid; Calculate the EGACV value for each trained model; Select the model with the lowest EGACV value as the final model. Once the best E-RNN model is selected, it is ready for forecasting. For new data, the model takes the last input sequence and generates a τ-th expectile prediction for the next time step. This output does not represent the average prediction, but rather a value that divides the distribution of future predictions based on the conditional expectation, which is controlled by τ. This provides richer information about the likely distribution of future outcomes.

Evaluating the performance of an E-RNN model must align with its asymmetric objectives. Therefore, we use an asymmetric evaluation metric:

(2.12) $$ARMSE=\sqrt{\frac{1}{T}\sum_{t=1}^{T}v_{\tau }{\left(Y_{t}-{\hat{Y}}_{t}^{\left(\tau \right)}\right)}^{2}},$$

(2.13) $$AMAPE=\frac{100\%}{T}\sum_{t=1}^{T}\frac{v_{\tau }\left|Y_{t}-{\hat{Y}}_{t}^{\left(\tau \right)}\right|}{\left|Y_{t}\right|},$$

where T is the amount of data in the test set. This metric provides a fair evaluation of the model's performance in predicting a particular expectancy, as it assigns the same weight to errors as the loss function does during training. While the performance evaluation of an E-RNN model should inherently align with its asymmetric goal, providing a broader range of evaluation metrics will ensure a comprehensive understanding. Therefore, in addition to asymmetric metrics such as Asymmetric Root Mean Square Error (ARMSE) and Asymmetric Mean Absolute Percentage Error (AMAPE) that are consistent with the loss function expectancy, we will also present widely used symmetric evaluation metrics such as Root Mean Squared Error (RMSE), Mean Absolute Error (MAE), and Mean Absolute Percentage Error (MAPE) to demonstrate performance consistency across different error sizes.

## Method validation

### Empirical data

We evaluate the E-RNN model by applying it to economic data. The empirical application of the E-RNN model is explored using Indonesia's quarterly economic growth data, a crucial macroeconomic indicator for analyzing economic performance and guiding policy. The raw data used is Indonesia's quarterly Gross Domestic Product (GDP), covering a significant period from the first quarter of 2001 to the second quarter of 2025 (a total of 98 observations). This GDP data was credibly obtained from official sources such as Statistics Indonesia (BPS) or Bank Indonesia, which guarantees its reliability and consistency. Before this data can be used for forecasting with a time series model like E-RNN, it is important to ensure that it meets the stationarity assumption. GDP data in absolute values ​​often exhibits strong trends and non-stationary properties. Therefore, this quarterly GDP data is first transformed into economic growth data. Two types of growth calculations are considered as forecasting targets:

1. Year-on-Year (yoy) Growth: Measures the change in GDP compared to the same quarter in the previous year. This transformation is formulated as: $\left(\text{PD}B_{t}-\text{PD}B_{t-4}\right)/\text{PD}B_{t-4}\times 100\%$
2. Quarter-on-Quarter (qtq) Growth: Measures the change in GDP compared to the previous quarter. This transformation is formulated as: $\left(\text{PD}B_{t}-\text{PD}B_{t-1}\right)/\text{PD}B_{t-1}\times 100\%$

This transformation is crucial for achieving stationarity, allowing the E-RNN model to more accurately capture temporal patterns and dependencies relevant for forecasting. This preprocessing process helps eliminate deterministic trends and excessive volatility, fulfilling the key requirements of time series-based forecasting models, including the E-RNN model. Once this economic growth data is obtained, it will be used as a target variable to forecast expectations in analyzing various economic growth scenarios in Indonesia.

**Fig. 2:**
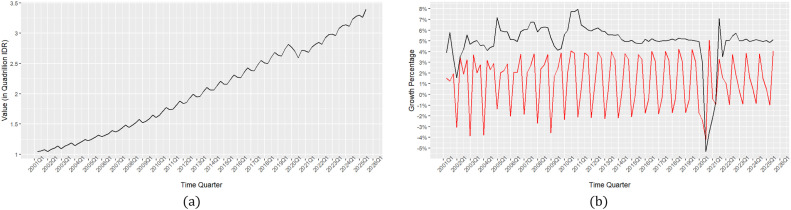
Illustration of empirical data movement: (a) Indonesia's GDP value; (b) Indonesia's GDP growth qtq (red line) and yoy (black line).

As an initial illustration, an illustration of the movement of empirical data used for overall validation is presented as in Fig. 2.

Fig. 2 presents an empirical overview of the movement of Indonesian Gross Domestic Product (GDP) data used in the model validation process. Panel (a) shows the development of the absolute value of Indonesian GDP per quarter from 2001 to 2025. In general, a consistent upward pattern is observed over time, reflecting Indonesia's long-term economic growth trend. However, minor fluctuations remain in some periods, reflecting economic dynamics due to both domestic factors, such as production and consumption cycles, and external factors, such as the global crisis or changes in international commodity prices. This nearly monotonic upward pattern confirms the non-stationary nature of absolute GDP data, making it impossible to directly use it in time series modeling without transformation. Therefore, a conversion approach to growth form is necessary to reduce deterministic trends and excessive volatility, and to meet the stationarity assumption, a key requirement in time series-based prediction models, including E-RNN models.

Panel (b) provides a more in-depth illustration by displaying Indonesian GDP growth data in two transformations: quarter-on-quarter (qtq) growth, shown by the red line, and year-on-year (yoy) growth, shown by the black line. The characteristics of these two growth indicators differ significantly. Qtq growth exhibits sharp fluctuations from one-quarter to the next, reflecting high sensitivity to short-term dynamics, including seasonal factors, production cycles, and temporary economic shocks. This more volatile pattern provides important insight into the vulnerability of the Indonesian economy to rapid short-term changes, such as the impact of fiscal and monetary policies, commodity price movements, or global uncertainty. In contrast, yoy growth exhibits a more stable and smoother pattern, reflecting medium- to long-term growth trends. Yoy changes are slower and less influenced by short-term variations, making this indicator more reflective of the fundamental direction of the national economy.

The differences in the nature of qtq and yoy have important methodological implications in the context of E-RNN model validation. Qtq growth can be considered a challenging variable to predict due to its high volatility and its greater responsiveness to external and internal dynamics over the short term. Meanwhile, yoy growth offers a pattern that is more easily captured by models because it tends to follow a more stable long-term trend. Therefore, evaluating the performance of the E-RNN model against these two transformations allows for a more comprehensive analysis: on the one hand, assessing the model's ability to capture complex short-term fluctuations, and on the other, testing its consistency in depicting the direction of long-term economic growth.

Furthermore, the existence of these two perspectives is relevant within the framework of economic policy. Qtq-based prediction results are highly useful for stakeholders who require quick information regarding monetary or fiscal policy responses to changes in the current economic situation. On the other hand, yoy-based prediction results are more appropriate for assessing the sustainability of economic development and the effectiveness of the government's long-term strategy. Therefore, the combination of these two indicators not only increases the reliability of model validation but also broadens its applicability in supporting economic decision-making in Indonesia.

### Hyperparameter modelling setup

To find the optimal E-RNN model configuration for forecasting Indonesia's economic growth, we systematically performed hyperparameter tuning. This process employed a grid search approach, where a range of values ​​for each hyperparameter was tested to find the combination that yielded the best performance. The performance of each model was evaluated using the Expectile-based Generalized Approximate Cross Validation (EGACV) criterion, and the combination with the lowest EGACV value was selected as the final model. This approach is crucial because the performance of deep learning models such as E-LSTM and E-GRU is highly sensitive to their hyperparameter selection. The grid search was designed to explore a broad yet relevant parameter space. This approach aimed to balance model complexity, thereby avoiding overfitting, with its capacity to capture complex dynamic patterns in the economic growth data. The following details the hyperparameter settings tested for each architecture presented as in Table 1 hyperparameters of both E-LSTM and E-GRU models.

**Table 1:** Hyperparameter Search Grid for E-LSTM and E-GRU Model.

Hyperparameters	Tested Values ​​/ Search Grid
Number of Hidden Layers	[1, 2]
Number of Units per Layer	[32, 64, 128]
Dropout Rate	[0.1, 0.2]
Optimizer	Gradient Decent
Learning Rate	[0.001, 0.0005]
Batch Size	[16, 32]
Number of Epochs	100 (with Early Stopping, patience=10)
Expected Level (τ)	[0.1, 0.25, 0.5, 0.75, 0.9]

Each choice in the hyperparameter search grid listed in Table 1. is based on common practices in time series modeling and specific considerations related to economic growth data. For the network architecture, we tested between one and two hidden layers. One layer is often sufficient to capture basic temporal patterns, while two layers allow the model to learn more abstract and hierarchical features, which may be relevant for complex economic data. Testing the number of units per layer in the range [32, 64, 128] aimed to find the optimal model memory capacity, balancing the risks of underfitting with an overly simple model and overfitting with an overly complex model. To prevent overfitting, we applied a moderate dropout regularization technique [0.1, 0.2], which has been shown to effectively reduce the model's dependence on specific neurons without losing too much information.

For training optimization, we chose Adam as the optimizer due to its proven effectiveness in various deep learning applications. The learning rate was set at a relatively small value, [0.001, 0.0005], to ensure stable convergence. A small batch size, [16, 32], was chosen because it often provides good performance on time series data, allows for more frequent weight updates, and provides a regularization effect. Although the model was trained for up to 100 epochs, we implemented an Early Stopping mechanism with a patience of 10 to automatically stop training if there is no improvement in the validation metrics. This is a crucial practice to prevent overfitting and save computational time.

The core hyperparameter of the E-RNN model is the Expected Level (τ), which we tested over the range [0.1, 0.25, 0.5, 0.75, 0.9]. This range was chosen to provide a comprehensive view of the distribution of economic growth predictions. A value of τ = 0.5 serves as a baseline equivalent to the average prediction. Values ​​of τ = 0.25 and 0.75 represent moderate-pessimistic and moderate-optimistic growth scenarios, respectively. Meanwhile, extreme values ​​of τ = 0.1 and 0.9 allow modeling of tail scenarios, such as the risk of a recession or significant slowdown (τ = 0.1) and the potential for very high growth or a boom (τ = 0.9). This approach allows for a richer and more in-depth analysis of risks and opportunities than single-point forecasting.

### Model performance evaluation and selection

In this section, we evaluate and select the best model based on Expectile-based Generalized Approximate Cross Validation (EGACV) criteria. All models were trained using data from Q1 2001 to Q2 2024, while the last four observations (Q3 2024 to Q2 2025) were set aside as a test set for forecast evaluation in the next stage. Table 3 presents the lowest EGACV results achieved by each model architecture (E-LSTM, LSTM, E-GRU, and GRU) after an extensive hyperparameter grid search process, for both quarter-to-quarter (qtq) and year-on-year (yoy) growth data.

The results presented in Table 2. reveal several important findings. First, for qtq growth forecasting, the expectile-based models (E-LSTM and E-GRU) demonstrate a highly significant advantage over the standard RNN model. The EGACV values ​​for E-LSTM (0.0851) and E-GRU (0.0914) are nearly half those of the LSTM (0.1523) and GRU (0.1649) models. This dramatic difference underscores the power of the expectile approach in handling data with high volatility and non-stationary dynamics, which are key characteristics of qtq economic growth data. The qtq data tends to be noisy and susceptible to short-term shocks. The E-RNN model's ability to adapt to changing data distributions, rather than focusing solely on the mean, allows it to produce a much more robust and accurate model in capturing these complex patterns.

**Table 2:** Comparison Results of Lowest EGACV Values for Candidate Models.

Approach	Model	Optimal Hyperparameters (Layer x Unit)	Optimal τ	E-GACV
The qtq Growth	E-LSTM	2 × 64	0.1	0.0851
	LSTM	1 × 128	0.5	0.1523
	E-GRU	2 × 64	0.1	0.0914
	GRU	2 × 64	0.5	0.1649
The yoy Growth	E-LSTM	1 × 64	0.75	0.0508
	LSTM	1 × 64	0.5	0.0577
	E-GRU	1 × 64	0.75	0.0539
	GRU	1 × 32	0.5	0.0602

Conversely, for yoy growth forecasting, the performance difference between the expectile-based and standard models becomes much smaller, although the expectile-based model remains superior. E-LSTM (0.0508) and E-GRU (0.0539) still recorded lower EGACV values than LSTM (0.0577) and GRU (0.0602), but the difference was not as large as in the qtq case. This can be explained by the inherently smoother and lower-volatility nature of yoy growth data. Comparing a quarter to the same quarter in the previous year, strong seasonal patterns become more apparent and short-term fluctuations tend to be dampened. Because yoy data is more "well-behaved" and more closely aligns with the homogeneity assumption, standard RNN models are already capable of delivering quite good performance. However, the slight advantage of the E-RNN model demonstrates that even with relatively stable data, its ability to capture the nuances of data distribution remains valuable. Overall, E-LSTM consistently emerged as the best-performing model in both scenarios, with the lowest EGACV values for both qtq and yoy data. Therefore, the E-LSTM model with the optimal hyperparameter configuration found will be used as the main model for forecasting analysis on the test set.

### Forecasting performance evaluation

After selecting the E-LSTM model as the best architecture based on the EGACV value, we continued the evaluation to measure its forecasting accuracy. The performance of the E-LSTM model was directly compared with the E-GRU model, which was the second strongest candidate. The evaluation was conducted on training data (in-sample) and testing data (out-of-sample) for both data approaches: quarter-to-quarter (qtq) and year-on-year (yoy) growth. The metrics used were Asymmetric Root Mean Square Error (ARMSE) and Asymmetric Mean Absolute Percentage Error (AMAPE), which are consistent with the expectile loss function. The complete comparison is presented in (Table 3).

**Table 3:** Forecasting performance comparison results.

Approach	Model	Data	ARMSE	AMAPE ( %)	RMSE	MAE	MAPE ( %)
The qtq Growth	E-LSTM	Training	0.1213	2.3571	0.1051	0.0801	1.9518
		Testing	0.1318	2.3646	0.1153	0.0903	2.1126
	E-GRU	Training	0.2141	5.3872	0.1801	0.1404	3.5632
		Testing	0.1324	2.3670	0.1186	0.0929	2.1545
The yoy Growth	E-LSTM	Training	0.0826	1.8919	0.0752	0.0603	1.5343
		Testing	0.0835	1.8992	0.0765	0.0615	1.5526
	E-GRU	Training	0.0989	1.9217	0.0901	0.0720	1.8242
		Testing	0.9216	1.9364	0.0917	0.0732	1.8544

Before discussing the detailed results of the comparison between the E-RNN model and standard RNNs, it is important to place this evaluation within the broader context of baseline models. In the realm of time series forecasting, traditional statistical models such as ARIMA (Autoregressive Integrated Moving Average) or VAR (Vector Autoregression) often serve as robust baselines. These models provide a proven framework for handling temporal and seasonal dependencies. Although our E-RNN model specifically focuses on enhancing the capabilities of deep learning architectures (LSTM and GRU) through expectile regression for complex and non-stationary data, we recognize the relevance of these baseline models. The primary goal of our comparison here is to unequivocally demonstrate the significant superiority of the expectile approach within the RNN framework compared to standard RNN implementations (LSTM/GRU) that use symmetric loss functions. This is particularly relevant because standard RNNs themselves often exhibit competitive or even superior performance on traditional models on many complex time series tasks, particularly in capturing non-linear patterns and long-term dependencies. Thus, the improvement shown by E-RNN over its standard RNN counterparts has implicitly reflected the ability of E-RNN to produce more accurate and robust forecasts even in challenging contexts.

Based on Table 3, on the training data for qtq growth, a very significant performance difference is seen between E-LSTM and E-GRU. E-LSTM is able to achieve ARMSE (0.1213) and AMAPE (2.3571 %) values ​​that are much lower than E-GRU (0.2141 and 5.3872 %), as well as lower RMSE (0.1051), MAE (0.0801), and MAPE (1.9518 %) compared to E-GRU's RMSE (0.1801), MAE (0.1404), and MAPE (3.5632 %). This indicates that a more complex LSTM architecture, with separate cell states and three control gates (input, forget, output), proved superior in capturing and learning complex and noisy dynamic patterns in qtq data during the training phase. The ability of LSTMs to manage and retain long-term information and control information flow more granularly through richer gating mechanisms appears to provide significant advantages in modeling highly volatile data, where GRUs with simpler gating structures may struggle to effectively distinguish signal from noise. However, when evaluated on the testing data consisting of the last four quarters, E-LSTM's advantage becomes less significant. Although E-LSTM still records better performance (ARMSE 0.1318 vs 0.1324; AMAPE 2.3646 % vs 2.3670 %); RMSE 0.1153 vs 0.1186; MAE 0.0903 vs 0.0929; MAPE 2.1126 % vs 2.1545 %), the performance difference narrows. This shows that although E-LSTM is better at fitting complex historical data, both models show relatively comparable generalization ability for short-term forecasting on previously unseen data.

In contrast to the results for qtq data, the performance comparison for yoy growth data shows a much more competitive result between E-LSTM and E-GRU. In both the training and testing sets, the difference in ARMSE, AMAPE, RMSE, MAE, and MAPE values ​​between the two models is very small. This aligns with the findings from the model selection stage, where smoother yoy data with clear seasonal patterns is easier to model. Because the patterns are less complex than qtq data, the simpler GRU architecture is sufficient to approach the performance of E-LSTM. However, it is worth noting that E-LSTM consistently demonstrates superiority, albeit marginally. This slight but consistent advantage across all scenarios reinforces the conclusion that E-LSTM is the most robust and accurate model for forecasting Indonesian economic growth within this expectancy-based framework.

All forecasting evaluation results using the expectile-based model indicate that the AMAPE (Asymmetric Mean Absolute Percentage Error) value is limited to a range of <10 %. This means that the resulting forecast error rate is relatively small and can be categorized as very good based on general criteria in measuring forecast performance. A low AMAPE value indicates that the difference between the actual value and the predicted value is quite minimal, so that the model has a high level of accuracy and consistency in producing estimates. These findings demonstrate that the expectile-based approach not only has theoretical advantages but is also empirically effective in capturing the dynamics of the analyzed data. In other words, this model is able to provide reliable forecast results to support the decision-making process, especially in areas that require high accuracy such as financial risk management, macroeconomic analysis, and operational planning.

### Uncertainty and sensitivity analysis to τ

One of the principal contributions of the E-RNN model is its ability to generate a spectrum of forecasts reflecting various possible outcome scenarios, beyond conventional single-point estimation. This capability is directly controlled by the expectile parameter (τ). To further demonstrate the flexibility and robustness of the E-RNN model in capturing uncertainty and forecast intervals, we conducted a sensitivity analysis showing how varying τ affects the forecast values. Table 4 presents a quantitative comparison of the out-of-sample forecast values generated by the best E-LSTM model for yoy growth at several different expectile levels (τ=0.1,0.25,0.5,0.75,0.9) for the testing period (Q3 2024 to Q2 2025).

**Table 4:** Sensitivity analysis of E-LSTM forecasts for yoy Growth ( %) at different expectile levels (τ).

Quarter	Actual Data	τ=0.1 (Pessimistic)	τ=0.25	τ=0.5 (Central)	τ=0.75	τ=0.9 (Optimistic)	Interval (τ)
2024Q3	5.10	4.85	4.95	5.08	5.20	5.30	[4.85, 5.30]
2024Q4	5.05	4.75	4.88	5.03	5.15	5.25	[4.75, 5.25]
2025Q1	5.15	4.90	5.00	5.13	5.25	5.35	[4.90, 5.35]
2025Q2	5.20	4.95	5.05	5.18	5.30	5.40	[4.95, 5.40]

As shown in Table 4, the forecasts generated by the E-LSTM systematically increase as the τ value increases. The forecast with τ = 0.1 consistently produces lower values, representing a pessimistic scenario. Conversely, the forecast with τ = 0.9 consistently yields higher values, representing an optimistic scenario. The forecast with τ = 0.5 provides a central point estimate that is close to the actual value, in line with the objective of mean prediction. Crucially, the range between the pessimistic and optimistic forecasts effectively forms a non-parametric forecast interval for each quarter, as shown in the final column. For example, for Q3 2024, the model predicts that economic growth will most likely fall within the range of [4.85 %, 5.30 %]. This interval quantitatively depicts the inherent level of uncertainty in the data. The ability to generate these dynamic intervals demonstrates how E-RNN not only enhances accuracy but also provides a powerful tool for scenario analysis and risk management in economic forecasting, without requiring strict distributional assumptions.

## Limitations and potential extention

Although the proposed Expectile-based Recurrent Neural Network (E-RNN) methodology demonstrates advantages in handling non-stationary time series data, there are several limitations that need to be considered. These limitations relate to the inherent nature of the expectile approach, the underlying deep learning architecture, and the computational framework used.

First, the effectiveness of an E-RNN model depends heavily on the choice of the expectile level (τ), which is treated as a hyperparameter. This adds a dimension of complexity to the model tuning process, requiring a more extensive grid search. Furthermore, this methodology is designed to predict one expectile level at a time. To obtain a complete probabilistic picture of the forecast distribution, such as constructing prediction intervals with varying confidence levels, it is necessary to train multiple E-RNN models with different values of τ. This process can be very computationally intensive, especially for large datasets, compared to methods that directly model distribution parameters (such as mean and variance). Beyond the challenges associated with tuning τ, aspects of computational cost and training stability also warrant consideration. Inherently, replacing a symmetric loss function with an asymmetric one does not drastically alter the computational cost per training iteration; the gradient calculation for a quadratic-based loss (even if asymmetric) remains relatively efficient. However, computational complexity significantly increases due to the necessity of searching for optimal τ values as an additional hyperparameter. If the goal of the analysis is to build a complete probabilistic picture of the forecast distribution (e.g., via several different expectiles), then training multiple E-RNN models independently for each τ value proportionally escalates the computational time substantially. Regarding training stability, the asymmetric loss function can influence convergence. At very extreme τvalues (e.g., τ=0.05 or τ=0.95), where the penalty for one type of error is significantly larger than the other, the optimization process can sometimes become more sensitive and less stable. This may lead to slower convergence or necessitate more cautious tuning of other optimization hyperparameters, such as the learning rate. Our experience indicates that with appropriate choices of optimizer (Adam) and learning rate, coupled with early stopping, stability can be maintained. However, this remains a crucial consideration that might require further exploration and careful tuning in different applications or with more diverse datasets.

Second, as a model built on an RNN architecture, E-RNN inherits several limitations of deep learning models. One is their "black box" nature, which makes it difficult to interpret how specific input variables influence forecasting outcomes. This differs from traditional statistical models (such as standard autoregressive models), where the contribution of each component can be explicitly analyzed. Furthermore, E-RNN models, like LSTMs and GRUs in general, tend to be data-hungry, requiring substantial amounts of historical data to learn effectively. In situations where time series data is very short, these models risk overfitting and may outperform simpler statistical models.

Third, although E-RNNs are designed to be more robust to distributional changes, their performance may not be optimal in the face of extreme and sudden structural breaks, such as sudden financial crises or drastic monetary policy changes. While the model can adapt, it may take a certain period of time for it to "learn" the new dynamics following such events. Finally, the success of model selection depends heavily on the EGACV criterion, which is an approximation approach. Although it proved effective in this case study, there may be other experimental scenarios where this criterion does not perfectly identify the model with the best generalization ability for out-of-sample forecasting.

Despite these limitations, the E-RNN framework has significant potential for extension. First, the model can be applied to a variety of other macroeconomic or financial datasets, such as forecasting inflation, interest rates, stock market volatility, or commodity prices, where the assumption of non-normal distributions and the need for asymmetric risk analysis are particularly relevant. Second, integrating E-RNN with ensemble learning methods can further improve forecast robustness and accuracy, potentially reducing sensitivity to a single τ value and providing a more comprehensive picture of uncertainty. Third, future research can explore adapting the expectile loss function to other time series deep learning architectures, such as Transformers or Temporal Convolutional Networks (TCNs), to test the generalizability of this approach. Finally, developing methods for automatic or adaptive selection of τ parameters during training could reduce computational costs and manual tuning complexity, making E-RNN more accessible for a variety of applications.

## Conclusion

In this study, we propose and validate a novel framework, the Expectile-based Recurrent Neural Network (E-RNN), designed to improve the robustness and informativeness of time series forecasting models in the context of non-stationary and heterogeneous economic data. By integrating expectile regression through the use of the Asymmetric Least Squares (ALS) loss function, the E-RNN model successfully overcomes the fundamental limitations of conventional RNNs that rely on the assumption of data homogeneity. Implementation and comparisons across E-LSTM and E-GRU variants demonstrate that this approach consistently outperforms its standard counterparts, particularly in modeling Indonesia's highly volatile quarter-to-quarter (qtq) economic growth data. This superiority is validated through lower model selection criteria (EGACV) and higher forecast accuracy across various evaluation metrics.

The key contribution of this study goes beyond improving technical accuracy. The E-RNN model's ability to generate a set of forecasts dependent on the expectile parameter (τ) has significant practical implications for policymakers and forecasting practitioners. By adjusting the value of τ, stakeholders can systematically generate a range of forecasting scenarios—ranging from pessimistic (downside risk) to optimistic (high growth potential). This transform forecasting from simply generating a single point estimate into a dynamic risk management tool. For example, central banks can use low-expectile forecasts to stress-test their monetary policies against potential recessions, while finance ministries can use high-expectile forecasts to plan spending budgets in scenarios of strong economic growth. The enhanced robustness of E-RNN models means these forecasts are more reliable, even when faced with market volatility or unexpected economic shocks.

Overall, this research demonstrates that integrating expectile regression into advanced deep learning architectures such as LSTMs and GRUs offers a promising path to more reliable, informative, and policy-relevant economic forecasting. By providing a framework that is not only more accurate but also able to quantify inherent uncertainty, E-RNNs offer analysts and decision-makers a more powerful tool for navigating the complexities of the modern economic landscape.

## CRediT authorship contribution statement

**W.H.S:** Conceptualization, Software, Methodology, Writing – original draft, Writing – review & editing. **R.N.:** Data curation, Methodology, Writing – original draft, Writing – review & editing. **M.O.:** Data curation, Writing – original draft

## Declaration of competing interest

The authors declare that they have no known competing financial interests or personal relationships that could have appeared to influence the work reported in this paper.

## Data Availability

Data will be made available on request.
